# Adherence to recommendations for nutrient supplementation related to pregnancy in Germany

**DOI:** 10.1002/fsn3.3482

**Published:** 2023-06-11

**Authors:** Berin Doru, Nele Hockamp, Erika Sievers, Philipp Hülk, Thomas Lücke, Mathilde Kersting

**Affiliations:** ^1^ Research Department of Child Nutrition University Hospital of Pediatrics and Adolescent Medicine, St. Josef‐Hospital, Ruhr‐University Bochum Bochum Germany; ^2^ Haale Germany

**Keywords:** dietary supplements, folic acid, iodine, lactation, micronutrients, pregnancy

## Abstract

Supplementation of certain micronutrients is recommended to ensure their adequate supply during pregnancy and lactation. In Germany, this applies particularly to folic acid and iodine. There is no nationwide data on adherence to the supplementation guidelines. The aim of this cross‐sectional study was to determine the prevalence and predictors of the recommended supplementation of both folic acid and iodine in mothers of a nationwide birth cohort. Data on supplementation, before, during, and shortly after pregnancy, were collected retrospectively 14 days postpartum in a sample of 962 mother–infant pairs participating in the second nationwide study on breastfeeding and infant nutrition in Germany, called “SuSe II” (2017–2019). Folic acid and iodine supplementation were classified as recommended according to the German guidelines if supplementation was provided for both essential periods: for folic acid before and during pregnancy and for iodine during pregnancy and lactation. Univariable tests and multivariable logistic regression analysis were performed. The vast majority of mothers did not adhere to the recommendations, with only 36.2% supplementing folic acid and 31.9% supplementing iodine during the recommended periods, and only 15.2% adhering to the recommendations for both nutrients. Main predictors of adherence to recommendations of both nutrients were lifestyle attributes and nutrition‐related intentions like previous breastfeeding experience and breastfeeding intentions, but not common sociodemographic characteristics. The data suggest widespread dissemination of the time‐specific recommendations covering the entire period from preconception to lactation that could help to sensitize women and healthcare providers.

## Graphical abstract

As part of a prospective nationwide breastfeeding survey mothers were asked 14 days postpartum about their supplementation of folic acid and iodine before and during pregnancy and lactation. While about a third of women supplemented folic acid or iodine as recommended, only about 15 % supplemented both. Among the significant predictors of adherence are maternal lifestyle attributes and nutrition‐related intentions rather than the common sociodemographic factors.

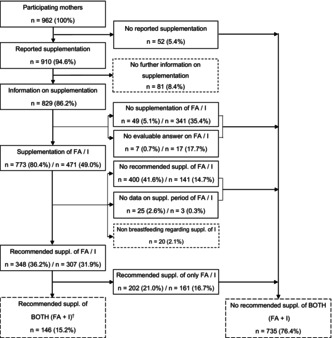

## 1 INTRODUCTION

The need for specific micronutrients during pregnancy and lactation is increased (Gernand et al., 2016; Koletzko et al., 2013; Pietrzik et al., 1997). International guidelines recommend that expectant mothers generally supplement folic acid, iron, and additionally iodine if the usual dietary iodine intake is inadequate (Gernand et al., 2016; Hanson et al., 2015; Tsakiridis et al., 2020; WHO, 2016). In Germany, prophylactic iron supplementation is generally limited to cases of medically diagnosed deficiency because of the potential risks of excessive iron concentrations (Deutsche Gesellschaft für Ernährung, 2018; Koletzko et al., 2018), whereas for Germany as an area of mild‐to‐moderate iodine deficiency (Esche et al., 2020; Hey et al., 2019; Ittermann et al., 2020; Johner et al., 2016; WHO, 2013), iodine supplementation is recommended in pregnancy and lactation (Deutsche Gesellschaft für Ernährung, 2018; Koletzko et al., 2018).

The increased demand for folic acid results from an increase in cell division and erythropoiesis, while the increased demand for iodine is due to a higher basal metabolic rate, and an increase in maternal production of thyroid hormones and renal iodine excretion during pregnancy (Bailey & Gregory 3rd., 1999; Bailey, 2000; Bung et al., 1996; Glinoer, 2007; Harding et al., 2017; Smyth et al., 1997). Possible consequences of folic acid deficiency include malformations, neural tube closure disorders, and cleft lip and palate (Czeizel & Bánhidy, 2011; Czeizel et al., 2013; Koletzko & von Kries, 1995; Shaw et al., 1995; Wilcox et al., 2007), while iodine deficiency can lead to an increased risk of postnatal mortality and malformations, hypothyroidism, and cognitive development disorders (Czeizel & Bánhidy, 2011; Dunn & Delange, 2001; Haddow et al., 1999; Harding et al., 2017; Klett et al., 1999; Pop et al., 1999; Remer et al., 2010).

One option for ensuring an adequate supply of essential micronutrients is food fortification, especially applied in the United States and Australia (Global Fortification Data Exchange, 2022). In contrast to other countries, mandatory fortification has not become established in Germany and is currently voluntary for both iodine and folic acid (Bundesinstitut für Risikobewertung, 2021a, 2021b; Gärtner et al., 2021; Global Fortification Data Exchange, 2022). Only in the former German Democratic Republic was iodine fortification of salt mandatory at the beginning of the 1980s (Lux & Walter, 2005; Meng & Scriba, 2002; Scriba et al., 2007). While the fortification of salt with iodine had its beginnings in the 1920s in Switzerland and the United States, it was introduced relatively late in Germany, namely in 1959, initially as a dietary food (Habermann et al., 1975; Kimball & Marine, 1992; Leung et al., 2012; Lux & Walter, 2005; Scriba et al., 2007). Additional fortification of iodized salt with folic acid was introduced in the early 2000s (Bissinger et al., 2018; Lux & Walter, 2005). In Germany, the Working Group on Iodine Deficiency (“Arbeitskreis Jodmangel”, AKJ), founded in 1984, and the Working Group on Folic Acid (“Arbeitskreis Folsäure & Gesundheit”, AKF), founded in 2002, that try to promote prophylactic measures, report that the market shares of iodized household salt with and without folic acid remained stable since the 2000s at between 70 and 80% of total table salt sales to households (AKF, 2023; AKJ, 2023; Großklaus, 2017; Obeid et al., 2016). In contrast, the market shares of iodized salt were between 20 and 30% of total table salt sales in bulk containers in Germany (Großklaus, 2017). The fortification dosage of salt with iodine is limited to 25 mg per kg and folic acid to 100 μg per g (Bundesinstitut für Risikobewertung, 2021a, 2021b; Gärtner et al., 2021; Weißenborn, 2005). Fortification of other foods such as flour and cereals with folic acid is also voluntary in Germany and not mandatory, as in the United States, for example, for flour or cereal‐grain products since 1998 (Bundesinstitut für Risikobewertung, 2021a, 2021b; Global Fortification Data Exchange, 2022; Kimball & Marine, 1992; Leung et al., 2012). The Federal Institute for Risk Assessment in Germany (“Bundesinstitut für Risiobewertung,” BfR) pleads for a stronger focus on educational campaigns about the increased need for folic acid and iodine during pregnancy and appropriate individual supplementation (Bundesinstitut für Risikobewertung, 2021a, 2021b). Consequently, optimal supply for expectant mothers in Germany currently depends to a large extent on adherence to national recommendations on supplementation. These have been published for about 10 years by the “Healthy Start – Young Family Network,” authorized by the Federal Ministry of Food and Agriculture (BMEL) (Koletzko et al., 2013, 2018). In addition to a balanced and varied diet, daily supplementation of 400 μg folic acid is recommended from the time pregnancy is planned or at least 1 month before conception up to 12 weeks of pregnancy or 800 μg folic acid if supplementation was started less than 4 weeks before conception (Bundesinstitut für Risikobewertung, 2021a, 2021b; Koletzko et al., 2018). For iodine, a dose of 10–150 μg per day is recommended during both pregnancy and lactation (Bundesinstitut für Risikobewertung, 2021a, 2021b; Koletzko et al., 2018).

Several studies from the European area report insufficient folic acid supplementation of expectant mothers while iodine supplementation is often not considered (Birkenberger et al., 2019; Blumfield et al., 2013; Dante et al., 2015; Fulford et al., 2014; TEDDY study, 2013; Wegner et al., 2020). Thus, it is necessary to assess the extent to which the recommendations formulated for Germany are being followed.

Therefore, the objective of the present study was to analyze data on supplementation of mothers from a large birth cohort in Germany, namely the second nationwide study on breastfeeding and infant nutrition (in German: Stillen und Säuglingsernährung), called “SuSe II” (2017–2019), regarding the adherence to the supplementation guidelines related to pregnancy and lactation. In addition, potential predictors of adherence to supplementation recommendations were explored.

## 2 METHODS

### 2.1 Study design

The study on “Breastfeeding and infant nutrition in Germany,” named “SuSe II” (2017–2019), is the second nationwide study that combined a cross‐sectional survey on breastfeeding promotion in hospitals with a prospective survey of mother–infant pairs recruited in the participating hospitals. Five follow‐up assessments were scheduled at 0.5, 2, 4, 6, and 12 months after birth. All data were collected via web‐based questionnaires. The study was conducted according to the guidelines of the Declaration of Helsinki and all procedures were approved by the Ethics Committee of the Medical Faculty of the Ruhr University Bochum. Written informed consents were obtained from hospitals and mothers. A detailed description of the study design was published earlier (Hockamp et al., 2021).

### 2.2 Data assessments

The present analysis focuses on the supplementation of the two essentially critical nutrients folic acid and iodine in the maternal cohort before and during pregnancy as well as during early lactation. These data were collected at the first regular assessment 14 days postpartum (pp) as follows: “Did you take nutrient supplements (e.g. folic acid, iron, vitamin B12, omega‐3 fatty acids, multivitamins, magnesium, iodine) during pregnancy?” (Answer options: yes/no). If the answer was yes, several text fields had to be filled in with the names of the supplements taken, either as individual nutrients such as folic acid or iodine or as combination preparations consisting of several nutrients, the multiple micronutrient supplements (MMS). Since such MMS may contain many different nutrients, the package inserts and ingredients of these preparations were consulted and reviewed as part of the data analysis to determine whether they contained folic acid and/or iodine and whether they contained at least the recommended dosage. In addition, mothers were requested to indicate the periods of intake of each preparation by first asking whether they had started the supplementation before pregnancy or from which week of pregnancy onwards, up to which week of pregnancy they had continued the intake or whether they were still taking it 14 days pp., that is, at the time of the assessment.

Folic acid intake was classified as “recommended” when reported in both recommended periods, before and during pregnancy. Iodine supplementation was classified as “recommended” when reported in both recommended periods, during pregnancy and lactation. Here, the lactation period corresponded to the 14 days pp. Mothers who reported taking iodine during pregnancy but no longer at 14 days pp, and also were not breastfeeding, were not included in the classification of adherence to iodine supplementation. When preparation names were given, adherence to the minimum recommended dose was also considered, 400 μg in the case of folic acid, and 100 μg in the case of iodine. Mothers who had supplemented both nutrients as recommended in the respective periods were assigned to the “adherent” group and the others to the “nonadherent” group.

In addition to the questions on nutrient supplementation, the assessment 14 days pp also included questions on sociodemographic factors, other prenatal and perinatal characteristics of mother and infant, and the infant's current feeding status.

### 2.3 Statistical analysis

To explore potential associations between supplementation habits and other maternal attributes, the following 12 maternal characteristics were analyzed: maternal age, academic qualification, employment before maternity leave, parity, relationship status, residential area in Germany, medication use, smoking during pregnancy, diet, intention to full breastfeeding, breastfeeding status 14 days pp, and sources of information on breastfeeding.

Data were analyzed using the software package IBM® SPSS® Statistics Version 25.0 for Windows 2016 (IBM Corp.). Frequencies and percentages were used to describe the categorical variables. Multicollinearity was tested to rule out that the variables correlate closely with one another; tolerance values >0.2 were assumed as acceptable. Fisher's exact test (two sided) was used to test for associations between maternal characteristics and adherence to folic acid and iodine supplementation recommendations, both together and separately. *P*‐values <.05 were considered significant.

To examine the association between maternal characteristics and adherence to supplementation recommendations with adjustment of results for confounding factors, odds ratios (ORs) and their corresponding 95% confidence intervals (CIs) were calculated using multivariable binary logistic regression analysis with variable selection through backward elimination according to the likelihood statistic. Complying with supplementation recommendations of folic acid and iodine was coded 1, not complying was coded 0 and used as the dependent variable in the regression analysis.

## 3 RESULTS

### 3.1 Study sample

**FIGURE 1:**
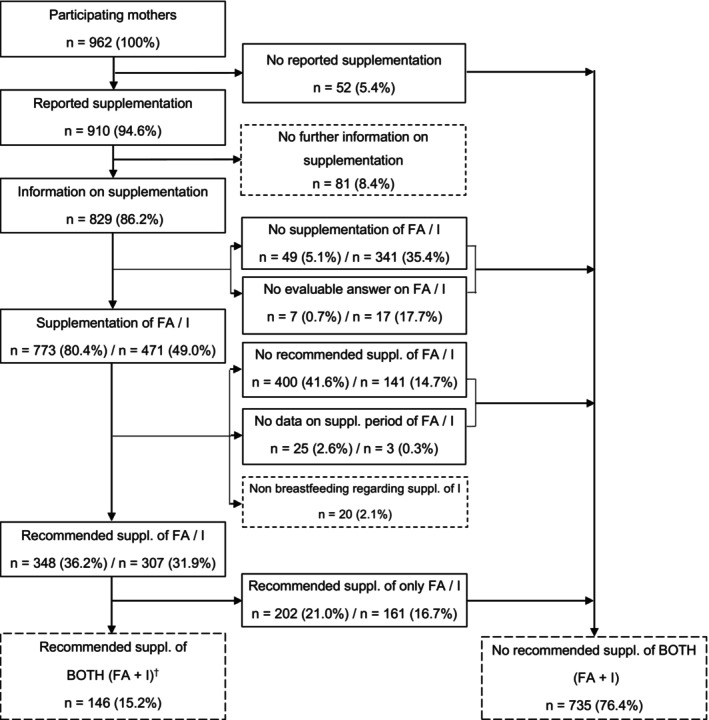
Flowchart of nutrient supplementation in SuSe II; FA, folic acid; I, iodine. ^†^1 of the 146 mothers did not breastfeed her infant 14 d pp but supplemented iodine.

Figure 1 provides an overview of the supplementation habits in the SuSe II cohort and the resulting samples, first separately for the recommended supplementation of folic acid and iodine, and finally, for both nutrients together, as defined in the final outcome. A total of 962 mothers in the SuSe II study completed the questionnaire 14 days pp (initial sample). Of these, 86.2% (*n* = 829) provided evaluable information on their supplementation. The data records could not be evaluated if supplementation was indicated but nutrient or preparation names were not specified or if the information on folic acid or iodine content could not be verified. Overall, folic acid was supplemented by 80.4% and iodine by 49.0% of the mothers in general, while 36.2% supplemented folic acid and 31.9% iodine as recommended, and 15.2% adhered to the recommendations for both nutrients.

### 3.2 Characteristics

For *n* = 81 participants, responses on folic acid and iodine supplementation could not be reliably evaluated. For all other participants (*n* = 881) descriptive characteristics are shown in Table 1. The proportion of mothers who reported being employed before maternity leave was slightly higher in the group of mothers supplementing both nutrients as recommended (adherent group) than in the larger group of mothers who did not supplement as recommended (nonadherent group) (88.4% vs. 81.0%; *p* = .033). About 40% of complying mothers lived in a more rural area, while the same proportion of noncomplying mothers stated living in an urban area. The proportion of mothers who took a medication besides nutrient supplements or who followed a vegetarian/vegan diet was higher in the adherent group than in the nonadherent group (24.1% vs. 16.2%; *p* = .031 and 10.3% vs. 4.1%; *p* = .006). Specific breastfeeding intentions, namely to breastfeed up to 4 or 6 months or longer than 6 months, were represented more often in the adherent group, whereas the proportion of mothers who were unsure whether or not to be able to breastfeed or who did not intend to breastfeed was higher in the nonadherent group (76.7% vs. 61.8% and 14.5% vs. 6.2%; *p* = .001). A higher proportion of complying mothers exclusively breastfed 14 days pp compared to noncomplying mothers (80.8% vs. 71.2%; *p* = .019). No significant associations between the adherence to supplementation recommendations and the following characteristics were found: maternal age, academic qualification, parity, relationship status, smoking during pregnancy, and most important information sources on breastfeeding.

**TABLE 1:** Characteristics of the participants regarding adherence to supplementation recommendations of both folic acid and iodine.

Characteristics	All participants (*n* = 881)	Adherent (*n* = 146)	Nonadherent (*n* = 735)	*p*‐value*
	*n* (%)	*n* (%)	*n* (%)	
*Age (years)* ^a^				
<30	192 (21.9)	23 (15.9)	169 (23.1)	.152
30–34	363 (41.3)	64 (44.1)	299 (40.8)	
≥35	323 (36.8)	58 (40.0)	265 (36.2)	
*Academic qualification* ^b^				
Low	60 (6.8)	5 (3.4)	55 (7.5)	.059
Medium	229 (26.1)	32 (21.9)	197 (26.9)	
High	588 (67.0)	109 (74.7)	479 (65.5)	
*Employment before maternity leave*				
Yes	724 (82.2)	129 (88.4)	595 (81.0)	**.033**
No	157 (17.8)	17 (11.6)	140 (19.0)	
*Parity*				
Primipara	444 (50.4)	79 (54.1)	365 (49.7)	.365
Multipara	437 (49.6)	67 (45.9)	370 (50.3)	
*Relationship status*				
Single parent	28 (3.2)	2 (1.4)	26 (3.5)	.297
Stable partnership	853 (96.8)	144 (98.6)	709 (96.5)	
*Residential area*				
Urban area	343 (38.9)	45 (30.8)	298 (40.5)	**.023**
Outer conurbation area	263 (29.9)	42 (28.8)	221 (30.1)	
Rural environment	275 (31.2)	59 (40.4)	216 (29.4)	
*Medication 14 days pp* ^c^				
Yes	154 (17.5)	35 (24.1)	119 (16.2)	**.031**
No	725 (82.5)	110 (75.9)	615 (83.8)	
*Smoking during pregnancy*				
Yes	50 (5.7)	4 (2.7)	46 (6.3)	.116
No	831 (94.3)	142 (97.3)	689 (93.7)	
*Diet*				
Omnivorous	836 (94.9)	131 (89.7)	705 (95.9)	**.006**
Vegetarian/vegan	45 (5.1)	15 (10.3)	30 (4.1)	
*Intention to full breastfeeding*				
As long as possible	199 (22.6)	25 (17.1)	174 (23.7)	.001
Up to 4 or 6 months/longer than 6 months	566 (64.2)	112 (76.7)	454 (61.8)	
Concerns whether it will work/no intention/not sure	116 (13.2)	9 (6.2)	107 (14.5)	
*Breastfeeding status 14 days pp*				
Exclusive breastfeeding	641 (72.8)	118 (80.8)	523 (71.2)	**.019**
Not exclusive breastfeeding	240 (27.2)	28 (19.2)	212 (28.8)	
*Most important information source— “breastfeeding”*				
By health personnel	203 (23.0)	32 (21.9)	171 (23.3)	.109
By social environment/other sources	218 (24.7)	41 (28.1)	177 (24.1)	
Previous breastfeeding experience/knowledge	336 (38.1)	61 (41.8)	275 (37.4)	
Not informed	124 (14.1)	12 (8.2)	112 (15.2)	

*Note*: Frequencies and percentages may not equal the total or may not add to 100% due to missing data.

Abbreviation: pp, postpartum.

^a^ Data of three participants are missing.

^b^ Data of four participants are missing; corresponding degrees: low‐ “Hauptschule/anderer Abschluss”: ≤9 years of schooling, medium‐ “Mittlere Reife mit Realschulabschluss”: ≤10 years of schooling, high‐ “Fachabi/Abi”: ≤11 years of schooling.

^c^ Data of 2 participants are missing; intake of medicaments besides nutrient supplements.

* Fisher exact *p*‐values significant and highlighted bold at <.05 comparing participants complying with folic acid and iodine supplementation recommendations with noncomplying participants.

In the Supplemental Material, descriptive characteristics of participants whose data could not be reliably evaluated and all other participants, are presented (Table S1) as well as the results of the comparisons of adherent and nonadherent participants separately for folic acid and iodine (Tables S2 and S3). Similar to the main examination of adherence to both nutrients (Table 1), some lifestyle characteristics, including breastfeeding, were also significant in the separate examinations. In addition, in the separate examinations, more relevant factors were related to sociodemographic characteristics such as age, academic qualification, parity, and relationship status (Tables S2 and S3).

### 3.3 Regression analysis

In the final model of the binary logistic regression analysis, seven variables were found to be significant regarding adherence to both folic acid and iodine supplementation recommendations. These are listed in Table 2 together with their (un‐) adjusted odds ratios and (un‐) adjusted confidence intervals. We refer to the adjusted results in the following section. Compared to mothers who were generally not informed about breastfeeding, mothers who had previous breastfeeding knowledge had 3.89 times higher odds of complying with supplementation recommendations of both nutrients (95% CI: 1.44, 10.52). A vegetarian/vegan diet compared to an omnivorous diet was associated with a higher likelihood of supplementing as recommended (OR: 2.67, 95% CI: 1.36, 5.25). Mothers with an intention to breastfeed for up to 4 months or longer had 1.66 times higher odds (95% CI: 1.02, 2.70) of supplementing as recommended compared to those who were unsure or not intended to breastfeed. In addition, primiparous mothers had 2.41 times higher odds (95% CI: 1.06, 5.50) of supplementing as recommended in comparison to multiparous mothers, and mothers who were employed before their maternity leave had 1.81 times higher odds (95% CI: 1.02, 3.20) than those who were not employed. Regular consumption of medications besides nutrient supplements was also associated with complying with supplementation recommendations (OR: 1.59, 95% CI: 1.02, 2.50). Compared to mothers who lived in an urban area, mothers who lived more rural had 1.81 times higher odds (95% CI: 1.16, 2.81) of supplementing both nutrients as recommended.

**TABLE 2:** Odds ratios for adherence to folic acid and iodine supplementation recommendations resulting from the multivariable binary logistic regression analysis.

Characteristics	Unadjusted			Adjusted		
	OR	CI	*p*‐value	OR	CI	*p*‐value
*Employment before maternity leave*						
No	1			1		
Yes	1.79	1.04, 3.06	**.035**	1.81	1.02, 3.20	**.041**
*Parity*						
Multipara	1			1		
Primipara	1.20	0.84, 1.71	.326	2.41	1.06, 5.50	**.037**
*Residential area*			**.022**			**.025**
Urban area	1			1		
Outer conurbation area	1.26	0.80, 1.98	.322	1.19	0.75, 1.91	.462
Rural environment	1.81	1.18, 2.77	**.006**	1.81	1.16, 2.81	**.008**
*Medication 14 days pp* ^a^						
No intake	1			1		
Intake	1.64	1.07, 2.52	**.023**	1.59	1.02, 2.50	**.042**
*Diet*						
Omnivorous	1			1		
Vegetarian/vegan	2.70	1.41, 5.14	**.003**	2.67	1.36, 5.25	**.004**
*Intention to full breastfeeding*			**.002**			**.015**
As long as possible	1			1		
Up to 4 or 6 months/longer than 6 months	1.72	1.08, 2.74	**.023**	1.66	1.02, 2.70	**.044**
Intention, but concerns whether it will work/no intention/not sure	0.59	0.26, 1.30	.189	0.70	0.31, 1.59	.388
*Most important information source—“breastfeeding”*			.134			**.052**
Not informed	1			1		
Previous breastfeeding experience/‐knowledge	2.07	1.07, 3.99	**.030**	3.89	1.44, 10.52	**.007**
By their social environment/other sources	2.16	1.09, 4.29	**.027**	1.81	0.89, 3.68	.101
By health personnel	1.75	0.86, 3.54	.121	1.53	0.74, 3.17	.249

*Note*: Not in the final model: maternal age, academic qualification, relationship status, smoking during pregnancy, and breastfeeding status 14 days pp. *p*‐values highlighted bold at <.05.

Abbreviations: CI, 95% confidence interval; OR, odds ratio; pp, postpartum.

^a^ Intake of medicaments besides nutrient supplements.

The results of the separate analysis for adherence to supplementation recommendations are shown for folic acid in Table 3 and for iodine in Table 4. Patterns of sociodemographic characteristics and lifestyle were quite similar in the combined and separate analyses, with sociodemographic characteristics more prominent in the separate analyses and parity particularly relevant in all three, suggesting that primiparous women were more likely to supplement the recommended nutrients than multiparous women. At least one of the breastfeeding parameters considered was significantly related to supplementation in the separate and joint regression analyses. In addition, the characteristic of smoking during pregnancy appeared to be significant in both separate analyses, with nonsmoking mothers more likely to supplement the appropriate nutrient as recommended.

**TABLE 3:** Odds ratios for adherence to folic acid supplementation recommendations resulting from the multivariable binary logistic regression analysis.

Characteristics	Unadjusted			Adjusted		
	OR	CI	*p*‐value	OR	CI	*p*‐value
*Academic qualification* ^a^			**.001**			**.034**
Low	1			1		
Medium	1.80	0.90, 3.63	.098	1.17	0.56, 2.45	.683
High	3.06	1.60, 5.92	**.001**	1.74	0.86, 3.53	.126
*Employment before maternity leave*						
No	1			1		
Yes	2.11	1.43, 3.11	**.001**	1.81	1.21, 2.73	**.004**
*Parity*						
Multipara	1			1		
Primipara	1.50	1.14, 1.97	**.004**	1.42	1.06, 1.89	**.018**
*Relationship status*						
Single parent	1			1		
Stable Partnership	0.17	0.05, 0.55	**.003**	0.20	0.57, 0.68	**.010**
*Smoking during pregnancy*						
Yes	1			1		
No	5.22	2.20, 12.41	**.001**	3.77	1.53, 9.29	**.004**
*Intention to full breastfeeding*			**.001**			**.008**
As long as possible	1			1		
Up to 4 or 6 months/longer than 6 months	1.95	1.37, 2.78	**.001**	1.67	1.15, 2.41	**.007**
Intention, but concerns whether it will work/no intention/not sure	1.05	0.64, 1.75	.839	1.04	0.61, 1.76	.889

*Note*: Not in the final model: maternal age, residential area, medication 14 days pp, diet, breastfeeding status 14 days pp, and most important information source—“breastfeeding”. *p*‐values highlighted bold at <.05.

Abbreviations: CI, 95% confidence interval; OR, odds ratio; pp, postpartum.

^a^ Corresponding degrees: low‐ “Hauptschule/anderer Abschluss”: ≤9 years of schooling, medium‐ “Mittlere Reife mit Realschulabschluss”: ≤10 years of schooling, high‐ “Fachabi/Abi”: ≤11 years of schooling.

**TABLE 4:** Odds ratios for adherence to iodine supplementation recommendations resulting from the multivariable binary logistic regression analysis.

Characteristics	Unadjusted			Adjusted		
	OR	CI	*p*‐value	OR	CI	*p*‐value
*Age (years)*			**.006**			**.003**
≥35	1			1		
30–34	0.69	0.51, 0.95	**.023**	0.61	0.44, 0.85	**.004**
<30	0.56	0.38, 0.82	**.003**	0.53	0.35, 0.81	**.003**
*Parity*						
Multipara	1			1		
Primipara	1.05	0.79, 1.39	.737	2.08	1.22, 3.54	**.007**
*Smoking during pregnancy*						
Yes	1			1		
No	2.85	1.31, 6.17	**.008**	2.37	1.07, 5.25	**.033**
*Breastfeeding status 14 days pp*						
Not exclusive breastfeeding	1			1		
Exclusive breastfeeding	1.63	1.17, 2.28	**.004**	1.46	1.03, 2.06	**.034**
*Most important information source—“breastfeeding”*			**.015**			**.004**
Not informed	1			1		
Previous breastfeeding experience/‐knowledge	1.63	1.03, 2.56	**.036**	2.37	1.26, 4.49	**.008**
By their social environment/other sources	1.75	1.08, 2.84	**.023**	1.73	1.05, 2.86	**.033**
By health personnel	1.05	0.64, 1.73	.845	1.03	0.62, 1.73	.901

*Note*: Not in the final model: academic qualification, employment before maternity leave, relationship status, residential area, medication 14 days pp, diet, intention to full breastfeeding. *p*‐values highlighted bold at <.05.

Abbreviations: CI, 95% confidence interval; OR, odds ratio; pp, postpartum.

## 4 DISCUSSION

### 4.1 Overview

This analysis of a nationwide birth cohort of mother–infant pairs allows a specific insight into pre‐ and postpartum maternal supplementation habits of the nutrients, folic acid, and iodine, which have been generally recommended in Germany for many years. Although a clear majority of mothers reported taking nutrient supplements in general, adherence to the nationwide recommendations for folic acid and iodine supplementation in the respective peri‐ and postconceptional phases as well as postpartum was rather low with 36.2% for folic acid and 31.9% for iodine, and even lower with 15.2% for both nutrients.

The examination of the two critical nutrients separately and together suggests that the broad spectrum of variables associated with supplementation is well reflected when the two nutrients are considered together. Interestingly, sociodemographic characteristics such as maternal age or educational status that are often associated with health behavior and relevant for the individual nutrients here, too, were found to be nonsignificant factors for complying with both folic acid and iodine supplementation recommendations. Rather, lifestyle characteristics and nutrition‐related intentions were significant.

Specifically, the likelihood of supplementing both nutrients as recommended was higher in the presence of prior breastfeeding experience, breastfeeding intention up to 4 months or longer, a vegan/vegetarian maternal diet, medication, primiparity, employment before maternity leave, and rural residence. The broad spectrum of relevant factors for supplementation of the two critical nutrients suggests developing a broad‐based set of measures to improve adherence in order to reach the target groups as widely as possible.

### 4.2 Characteristics for supplementation

Despite the heterogeneity of existing studies, for example, in terms of the definition of supplementation, duration of supplementation, and assessment methods, some meaningful comparisons with other studies in Germany and internationally are possible.

#### 4.2.1 Sociodemographic characteristics

Associations between nutrient supplementation in pregnancy and sociodemographic factors were analyzed in the international multicenter TEDDY Study in 2013, where a sample of 7326 mothers of six clinical research centers (USA, Sweden, Finland, Germany) screened for high‐risk HLA‐DQ genotypes was surveyed (Aronsson et al., 2013). Primiparous women were more likely to use supplements during pregnancy, which is consistent with our findings (Aronsson et al., 2013). In contrast to our joint examination, in TEDDY, older women (only in the United States and Sweden) and women with higher education were more likely to take supplements during pregnancy (Aronsson et al., 2013). These results appear to be partially consistent with the results of our separate analyses, in that women with higher academic qualification were more likely to supplement folic acid as recommended and women aged 30–35 were more likely to supplement iodine as recommended. However, it should be noted that the authors defined a supplementing participant as anyone who reported having taken at least one supplement at least once during pregnancy, and supplements taken due to medical condition or illness were also included (Aronsson et al., 2013).

Surveys on supplementation in pregnancy often focus on folic acid supplementation rather than iodine, which is probably due to current international supplementation guidelines (Gernand et al., 2016; Hanson et al., 2015; Tsakiridis et al., 2020; WHO, 2016). Blumfield et al. (2013) found the data on iodine supplementation to be insufficient for a reliable meta‐analysis. Furthermore, they concluded that the supplementation of folic acid, among other nutrients, was consistently reported as being below national recommendations (Blumfield et al., 2013). This conclusion is consistent with our finding of inadequate adherence to specific nutrient recommendations.

In a local sample of pregnant and postpartum women in Germany (Berlin), 90.7% reported taking folic acid at some point during pregnancy, but only 37.8% also took it before conception, as actually recommended (Birkenberger et al., 2019). This is consistent with our results as 36.2% of the SuSe II sample reported folic acid supplementation before conception, but also during pregnancy. In addition, in the Berlin sample, women were more likely to take folic acid before conception if their pregnancy was planned, if they were better educated, earned more money, and were older (Birkenberger et al., 2019). Our study did not examine whether the participants had planned the pregnancy or how much money they earned. The problem seems to be relevant for Germany, as the Federal Center for Health Education (Bundeszentrale für gesundheitliche Aufklärung, BZgA) reported a proportion of about one‐third of unplanned pregnancies in Germany in the years of the survey, namely 2011–2015 (Bundeszentrale für gesundheitliche Aufklärung, BZgA, 2016). In SuSe II, in contrast to TEDDY and the Berlin sample, the characteristics, age and academic qualification, were not significant variables for recommended nutrient supplementation of both folic acid and iodine, but employment before maternity leave per se and academic qualification were significant variables for recommended folic acid supplementation.

#### 4.2.2 Other maternal characteristics

In a cross‐sectional international survey, 45% of participating women from France, Germany, Poland, and Belgium who were pregnant or planning to become pregnant supplemented folic acid (Fulford et al., 2014). The results showed, among other things, that perceiving oneself to be healthy was associated with decreased odds of supplementing folic acid (Fulford et al., 2014). The authors concluded that mental models of susceptibility to pregnancy are key in adherence to supplementation guidelines and assumed that women who saw themselves as healthy felt less susceptible, “invulnerable mom” (Fulford et al., 2014). This assumption would be consistent with our finding that mothers who need to take medications regularly were more likely to take the recommended supplements, as it might be assumed that these mothers are especially concerned about their health and would be unlikely to perceive themselves as less vulnerable. In our sample, characteristics like maternal diet and smoking behavior might reflect health consciousness, but subsamples were too small for an analysis of differences in adherence to supplementation.

A recent cross‐sectional regional survey in Saxony, Germany (Wegner et al., 2020), comes closest to our study design, as they recruited mothers in maternity hospitals with a similar sample size, but focused solely on folic acid supplementation. The results showed that 91.2% of their participants reported having supplemented folic acid at some point during the advised pre‐ and postconceptional period, but 47.4% began supplementing after their pregnancy was established (Wegner et al., 2020). Similar to our collective (36.2%). Wegner et al. (2020) found a proportion of 41.5% of mothers supplementing as recommended. In addition to similar considerations on associations between sociodemographic variables such as parity and folic acid supplementation in the Saxon and our nationwide study, the Saxon study found that unplanned pregnancy or later pregnancy diagnosis, higher parity, and lack of awareness of the importance of folic acid for optimal pregnancy outcomes were associated with nonsupplementation of folic acid during the periconceptional period (Wegner et al., 2020). In contrast, SuSe II assessed more data on maternal characteristics such as employment, residential area, medication, diet, and intentions regarding breastfeeding. Taken together, these two recent surveys from Germany point to poor adherence to folic acid supplementation recommendations.

### 4.3 Interpretation

The current study illustrates that more than 90% of women have taken nutrient supplements of any type, but far fewer take the recommended nutrients and even fewer take them at the right time. The poorer adherence to the individual recommendations for supplementation of specific nutrients at the respective periods before, during, and after pregnancy only becomes apparent when the data on the specific periods of nutrient supplementation are considered. In fact, women usually start supplementing too late in the case of folic acid and stop supplementing too early in the case of iodine.

Therefore, it should be clearly communicated, especially by healthcare providers, that it is not sufficient to take any supplements at any given time, but that there are specific periods of increased need. Time‐specific recommendations should be disseminated.

Furthermore, it should be conveyed that, in contrast to international supplementation guidelines, iodine plays a central role in the German recommendations and should therefore not be neglected. Time‐specific recommendations should be disseminated first of all in the (pre‐) pregnancy period, especially for folic acid. In Germany, preventive gynecological care and support for women would be an obvious option, already in the family planning phase.

Furthermore, it should be conveyed that, in contrast to international supplementation guidelines, iodine plays a central role in Germany and should therefore not be neglected. The factors identified to predict adherence to both critical nutrients, such as parity or breastfeeding intentions could be considered in individual and public health information. The finding that multiparous women were less prone to supplement correctly suggests that the attention of health education should ensure that women are not lulled into a false sense of security by relying on experiences in preceding pregnancies. Even if they and their infants may not have faced immediate clinical deficiency symptoms, for example, of folic acid, potential later consequences, for example of early iodine deficiency remain.

In the current situation in Germany, where compulsory fortification as a public health measure is not to be expected and individual prophylaxis through education of women is the focus, targeted multimedia and multiprofessional information strategies seem to be particularly important. Specifically, a standardized schedule for the necessary supplementation in the respective periods could be created, which would facilitate the explanation and dissemination of recommendations to women planning a pregnancy.

### 4.4 Strengths & limitations

Although there are several studies on the general supplementation of nutrients concerning pregnancy, only a few studies also consider the aspect of adherence to the specific recommendations (Fulford et al., 2014; Tsakiridis et al., 2020; Wegner et al., 2020). In addition, most studies focus on the intake of folic acid, while iodine supplementation is also explicitly recommended in Germany (Birkenberger et al., 2019; Fulford et al., 2014; Wegner et al., 2020). Therefore, an important strength of our study is that not only adherence to the recommended supplementation periods but also both ‘critical’ micronutrients are considered, both individually and together. Further strengths include nationwide recruitment and the collection of not only sociodemographic but also various lifestyle‐related data, as this not only allows for generalizability of results but also considers the specific intentions of different women and their relationship to adherence.

As the SuSe II study did not focus on maternal nutrient supplementation, it was not inquired whether the pregnancy was planned, although according to the Federal Center for Health Education (BZgA), the proportion of unplanned pregnancies in Germany is about one‐third. This could have affected the adherence to folic acid recommendations. Other weaknesses of the study include the retrospective collection of data 14 days pp, and that the data were self‐reported online so no queries could be made. As this is an observational study, only associations can be examined but not causality of factors for nutrient supplementation habits.

## 5 CONCLUSION

The results of our study suggest partial information and adherence deficits regarding nutrient supplementation in the reproductive‐age population in Germany. Indeed, the prevalence of mothers supplementing according to the recommendations is clearly insufficient, with a good 30% for folic acid and iodine in separate examinations and even only 15% in a joint examination. Widespread dissemination of the time‐specific recommendations, covering the entire period from preconception to lactation, could help to raise awareness and improve adherence among women and healthcare providers.

## AUTHOR CONTRIBUTIONS

**Berin Doru:** Formal analysis (lead); writing – original draft (lead). **Nele Hockamp:** Formal analysis (supporting); supervision (equal); writing – review and editing (lead). **Erika Sievers:** Supervision (equal); writing – review and editing (equal). **Philipp Hülk:** Formal analysis (equal); writing – review and editing (supporting). **Thomas Lücke:** Funding acquisition (lead); writing – review and editing (equal). **Mathilde Kersting:** Conceptualization (lead); supervision (equal); writing – review and editing (equal).

## CONFLICT OF INTEREST STATEMENT

The authors declare that they do not have any conflict of interest. MK and ES were members of the German National Breastfeeding Committee between 1994 and 2021 (MK) and between 2008 and 2021 (ES).

## ETHICAL APPROVAL

This study was approved by the Ethics Committee of the Medical Faculty of the Ruhr University Bochum.

## INFORMED CONSENT

Written informed consent was obtained from all study participants.

## Supporting information


Table S1

## Data Availability

Data are available upon request from the authors.
