# Supplementary material for: Adherence to recommendations for nutrient supplementation related to pregnancy in Germany
Source: Food Sci Nutr. 2023 Jun 11;11(9):5236–47. doi: 10.1002/fsn3.3482 (PMC10494575; doi:10.1002/fsn3.3482)
Supplement: Supplementary file 1 — Table S1 [file FSN3-11-5236-s001.docx]

Supplemental Material

S1 Characteristics of participants in terms of evaluable information about nutrient supplementation

| Characteristics | All participants  (n = 962) | Information on supplementation  (n = 829) | Non evaluable information on supplementation/ no supplementation  (n = 133) |  |
| --- | --- | --- | --- | --- |
|  | n (%) | n (%) | n (%) | p-value* |
| Age (years)^†^   - <30 - 30-34 - ≥35 | 214 (22.3)  402 (41.9)  343 (35.8) | 171 (20.7)  345 (41.8)  310 (37.5) | 43 (32.3)  57 (42.9)  33 (24.8) | **0.003** |
| Academic qualification^‡^   - Low - Medium - High | 73 (6.8)  256 (26.1)  626 (67.1) | 52 (6.3)  208 (25.2)  567 (68.6) | 21 (16.4)  48 (37.5)  59 (46.1) | **0.001** |
| Employment before maternity leave   - Yes - No | 782 (81.3)  180 (18.7) | 688 (83.0)  141 (17.0) | 94 (70.7)  39 (29.3) | **0.001** |
| Parity   - Primipara - Multipara | 479 (49.8)  483 (50.2) | 419 (50.5)  410 (49.5) | 60 (45.1)  73 (54.9) | 0.263 |
| Relationship status   - Single parent - Stable Partnership | 30 (3.1)  932 (96.9) | 24 (2.9)  805 (97.1) | 6 (4.5)  127 (95.5) | 0.289 |
| Residential area   - Urban area - Outer conurbation area - Rural environment | 371 (38.6)  288 (29.9)  303 (31.5) | 322 (38.8)  252 (30.4)  255 (30.8) | 49 (36.8)  36 (27.1)  48 (36.1) | 0.460 |
| Medication 14 days pp^§^   - Yes - No | 166 (17.3)  794 (82.7) | 148 (17.9)  679 (82.1) | 18 (13.5)  115 (86.5) | 0.266 |
| Smoking during pregnancy   - Yes - No | 56 (5.8)  906 (94.2) | 41 (4.9)  788 (95.1) | 15 (11.3)  118 (88.7) | **0.008** |
| Diet   - Omnivorous - Vegeterian/ vegan | 917 (95.3)  45 (4.7) | 786 (94.8)  43 (5.2) | 131 (98.5)  2 (1.5) | 0.074 |
| Intention to full breastfeeding   - As long as possible - Up to 4 or 6 months / longer than 6 months - Concerns whether it will work / no intention / not sure | 224 (23.3)  605 (62.9)  133 (13.8) | 177 (21.4)  543 (65.5)  109 (13.1) | 47 (35.3)  62 (46.6)  24 (18.0) | **0.001** |
| Breastfeeding status  14 days pp   - Exclusive breastfeeding - Not exclusive breastfeeding | 689 (71.6)  273 (28.4) | 607 (73.2)    222 (26.8) | 82 (61.7)  51 (38.3) | **0.007** |
| Most important information source- “breastfeeding”   - By health personnel - By social environment/ other sources - Previous breastfeeding experience / - knowledge - Not informed | 224 (23.3)  235 (24.4)  363 (37.7)  140 (14.6) | 189 (22.8)    210 (25.3)    318 (38.4)  112 (13.5) | 35 (26.3)  25 (18.8)  45 (33.8)  28 (21.1) | 0.056 |

Abbreviations: pp, postpartum.

Frequencies and percentages may not equal the total or may not add to 100% due to missing data.

^*^ Fisher exact p-values significant at <0.05 comparing participants complying with folic acid and iodine supplementation recommendations with noncomplying participants.

^†^Data of 3 participants are missing

^‡^Data of 7 participants are missing; corresponding degrees: low- “Hauptschule/anderer Abschluss”: ≤ 9 years of schooling, medium- “Mittlere Reife mit Realschulabschluss”: ≤ 10 years of schooling , high- “Fachabi/Abi”: ≤ 11 years of schooling

^§^Data of 2 participants are missing; intake of medicaments besides nutrient supplements

S2 Characteristics of the participants regarding adherence to supplementation recommendations of folic acid

| Characteristics | All participants providing information on folic acid supplementation  (n = 849) | Adherent  (n = 348) | Non-adherent  (n = 501) |  |
| --- | --- | --- | --- | --- |
|  | n (%) | n (%) | n (%) | p-value* |
| Age (years)^†^   - <30 - 30-34 - ≥35 | 187 (22.1)  347 (41.0)  312 (36.9) | 63 (18.2)  153 (44.1)  131 (37.8) | 124 (24.8)  194 (38.9)  181 (36.3) | 0.059 |
| Academic qualification^‡^   - Low - Medium - High | 55 (6.5)  221 (26.2)  569 (67.3) | 12 (3.4)  74 (21.3)  262 (75.3) | 43 (8.7)  147 (29.6)  307 (61.8) | **0.001** |
| Employment before maternity leave   - Yes - No | 698 (82.2)  151 (17.8) | 307 (88.2)  41 (11.8) | 391 (78.0)  110 (22.0) | **0.001** |
| Parity   - Primipara - Multipara | 428 (50.4)  421 (49.6) | 196 (56.3)  152 (43.7) | 232 (46.3)  269 (53.7) | **0.004** |
| Relationship status   - Single parent - Stable Partnership | 28 (3.3)  821 (96.7) | 3 (0.9)  345 (99.1) | 25 (5.0)  476 (95.0) | **0.001** |
| Residential area   - Urban area - Outer conurbation area - Rural environment | 327 (38.5)  256 (30.1)  266 (31.3) | 130 (37.4)  104 (29.9)  114 (32.8) | 197 (39.3)  152 (30.3)  152 (30.3) | 0.738 |
| Medication 14 days pp^§^   - Yes - No | 148 (17.5)  700 (82.5) | 67 (19.3)  280 (80.7) | 81 (16.2)  420 (83.8) | 0.270 |
| Smoking during pregnancy   - Yes - No | 48 (5.7)  801 (94.3) | 6 (1.7)  342 (98.3) | 42 (8.4)  459 (91.6) | **0.001** |
| Diet   - Omnivorous - Vegeterian / vegan | 806 (94.9)  43 (5.1) | 325 (93.4)  23 (6.6) | 481 (96.0)  20 (4.0) | 0.111 |
| Intention to full breastfeeding   - As long as possible - Up to 4 or 6 months / longer than 6 months - Concerns whether it will work / no intention / not sure | 189 (22.3)  550 (64.8)  110 (13.0) | 58 (16.7)  255 (73.3)  35 (10.1) | 131 (26.1)  295 (58.9)  75 (15.0) | **0.001** |
| Breastfeeding status  14 days pp   - Exclusive breastfeeding - Not exclusive breastfeeding | 624 (73.5)  225 (26.5) | 266 (76.4)    82 (23.6) | 358 (71.5)  143 (28.5) | 0.114 |
| Most important information source- “breastfeeding”   - By health personnel - By social environment/ other sources - Previous breastfeeding experience / - knowledge - Not informed | 196 (23.1)  212 (25.0)  325 (38.3)  116 (13.7) | 88 (25.3)    95 (27.3)    124 (35.6)  41 (11.8) | 108 (21.6)  117 (23.4)  201 (40.1)  75 (15.0) | 0.164 |

Abbreviations: pp, postpartum.

Frequencies and percentages may not equal the total or may not add to 100% due to missing data.

^*^ Fisher exact p-values significant at <0.05 comparing participants adhering to folic acid supplementation recommendations with non-adhering participants.

^†^Data of 3 participants are missing

^‡^Data of 4 participants are missing; corresponding degrees: low- “Hauptschule/anderer Abschluss”: ≤ 9 years of schooling, medium- “Mittlere Reife mit Realschulabschluss”: ≤ 10 years of schooling , high- “Fachabi/Abi”: ≤ 11 years of schooling

^§^Data of 1 participants are missing; intake of medicaments besides nutrient supplements

S3 Characteristics of the participants regarding adherence to supplementation recommendations of iodine

| Characteristics | All participants providing information on iodine supplementation  (n = 858) | Adherent  (n = 307) | Non-adherent  (n = 551) |  |
| --- | --- | --- | --- | --- |
|  | n (%) | n (%) | n (%) | p-value* |
| Age (years)^†^   - <30 - 30-34 - ≥35 | 190 (22.2)  353 (41.3)  312 (36.5) | 55 (18.0)  119 (38.9)  132 (43.1) | 135 (24.6)  234 (42.6)  180 (32.8) | **0.006** |
| Academic qualification^‡^   - Low - Medium - High | 56 (6.6)  224 (26.2)  574 (67.2) | 14 (4.6)  73 (23.9)  219 (71.6) | 42 (7.7)  151 (27.6)  355 (64.8) | 0.075 |
| Employment before maternity leave   - Yes - No | 707 (82.4)  151 (17.6) | 255 (83.1)  52 (17.0) | 452 (82.0)  99 (18.0) | 0.779 |
| Parity   - Primipara - Multipara | 435 (50.7)  423 (49.3) | 158 (51.5)  149 (48.5) | 277 (50.3)  274 (49.7) | 0.776 |
| Relationship status   - Single parent - Stable Partnership | 26 (3.0)  832 (97.0) | 9 (2.9)  298 (97.1) | 17 (3.1)  534 (96.9) | 1.000 |
| Residential area   - Urban area - Outer conurbation area - Rural environment | 337 (39.3)  254 (29.6)  267 (31.1) | 113 (36.8)  87 (28.3)  107 (34.9) | 224 (40.7)  167 (30.3)  160 (29.0) | 0.211 |
| Medication 14 days pp^§^   - Yes - No | 151 (17.6)  705 (82.4) | 62 (20.3)  244 (79.7) | 89 (16.2)  461 (83.8) | 0.136 |
| Smoking during pregnancy   - Yes - No | 47 (5.5)  811 (94.5) | 8 (2.6)  299 (97.4) | 39 (7.1)  512 (92.9) | **0.005** |
| Diet   - Omnivorous - Vegeterian / vegan | 815 (95.0)  43 (5.0) | 286 (93.2)  21 (6.8) | 529 (96.0)  22 (4.0) | 0.074 |
| Intention to full breastfeeding   - As long as possible - Up to 4 or 6 months / longer than 6 months - Concerns whether it will work / no intention / not sure | 194 (22.6)  555 (64.7)  109 (12.7) | 65 (21.2)  213 (69.4)  29 (9.4) | 129 (23.4)  342 (62.1)  80 (14.5) | **0.049** |
| Breastfeeding status  14 days pp   - Exclusive breastfeeding - Not exclusive breastfeeding | 635 (74.0)  223 (26.0) | 245 (79.8)    62 (20.2) | 390 (70.8)  161 (29.2) | **0.004** |
| Most important information source- “breastfeeding”   - By health personnel - By social environment/ other sources - Previous breastfeeding experience / - knowledge - Not informed | 201 (23.4)  210 (24.5)  327 (38.1)  120 (14.0) | 59 (19.2)    86 (28.0)    128 (41.7)  34 (11.1) | 142 (25.8)  124 (22.5)  199 (36.1)  86 (15.6) | **0.014** |

Abbreviations: pp, postpartum.

Frequencies and percentages may not equal the total or may not add to 100% due to missing data.

^*^ Fisher exact p-values significant at <0.05 comparing participants adhering to iodine supplementation recommendations with non-adhering participants.

^†^Data of 3 participants are missing

^‡^Data of 4 participants are missing; corresponding degrees: low- “Hauptschule/anderer Abschluss”: ≤ 9 years of schooling, medium- “Mittlere Reife mit Realschulabschluss”: ≤ 10 years of schooling , high- “Fachabi/Abi”: ≤ 11 years of schooling

^§^Data of 2 participants are missing; intake of medicaments besides nutrient supplements
